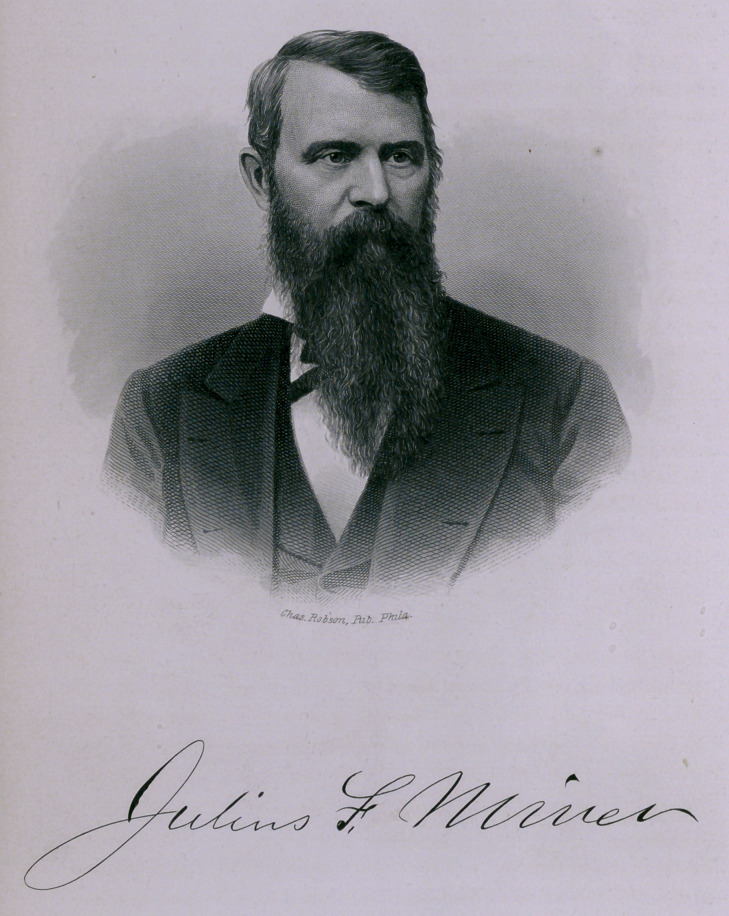# Julius F. Miner, M. D.

**Published:** 1886-12

**Authors:** 


Julius F. Miner

### Editorial.

### *JULIUS F. MINER, M. D.*

After a prolonged illness of several years, Dr. Miner died at his home in this city, Nov. 5th. He was beloved and respected by this community and by the medical profession. We can safely say that few physicians ever lived in this city who had the confidence of all to such a degree as did Dr. Miner. Even now after five years his place as a surgeon has hardly been filled; such skill and judgment as he possessed are rarely seen. As a teacher and an editor he won the plaudits of the profession. For many years he was the editor of this JOURNAL, and in addition to carefully prepared editoral matter, he added many original articles to its columns. Throughout his long and wearysome illness he was patient and hopeful. For over a year he had been quite comfortable, was able to read, and enjoyed hearing of affairs going on about him. When death at last came it was sudden and wholly unexpected. He passed away quietly, full of hope for the future life.

Dr. Miner was born in Peru, Berkshire Co., Mass., Feb. 16, 1823; educated at the Mountain Seminary, in Worthington, the Williston Seminary in East Hampton. He was graduated from the Berkshire Medical College, in Pittsfield, Mass., in 1846, and from the Albany Medical College in 1847. He came to Buffalo in 1855, where has since resided and practiced. In April, 1869, he made the first operation for ovariotomy by enucleation, and in June published the method of proceedure. He also ligated the external illiac for aneurism, successfully amputated at the hip joint; removed the thyroid gland for cystic bronchocele; removed popliteal aneurism by extirpation; removed the spleen, and performed most of the capital operations, full reports of which are found in the BUFFALO MEDICAL AND SURGICAL JOURNAL.

The Erie County Medical Society met, Nov. 7th to express in words the grief it felt in its loss. Dr. Rochester, a life-long friend, read a most appropriate memorial which is given below and contains many facts not already mentioned:

Julius F. Miner, M. D., passed quietly away, as the day was breaking on the 5th of November, 1886, in his sixty-fourth year. To the speaker this sad and significant event is most touching and impressive. It is the ending of an intimate social and professional friendship of over thirty years and of connection as hospital and college colleague for nearly as long a period. This experience, of more than a quarter of a century, is filled with bright and pleasing remembrance save only of the later period of sickness and suffering which he was called upon to endure, and even these could not quench or even obscure the enthusiasm and facetiousness with which he was most remarkably endowed. We are assembled here to honor his memory, and to pay this tribute of respect to which he was so fully entitled as a warm-hearted, generous man, and as a most distinguished and prominent and benevolent physician and surgeon. He came to this city in 1855, and so soon and so well did he establish himself, that he was made surgeon to the Buffalo General Hospital in 1860, subsequently the scene of so many of his brilliant and successful operations, and where he evolved and put in practice his system of enucleation for the removal of ovarian and other tumors, with which his name is associated the world over, and of which he received the most flattering recognition at the International Medical Congress, held in Philadelphia in 1876, where he expounded it, and attracted more attention and commendation than any other person on any other subject. He succeeded Prof. Sanford B. Hunt in 1861 as editor and proprietor of the BUFFALO MEDICAL AND SURGICAL JOURNAL, first established by Prof. Austin Flint, and conducted it for many years, until compelled by impaired health to transfer it to other hands. In 1867 he was elected to the chair of opthalmic and surgical anatomy in the medical department of the University of Buffalo, and in 1870 to that of special and clinical surgery, dividing the honors of the position with the eminent Prof. E. M. Moore of Rochester. In 1870 he was also made one of the surgeons to the Sisters of Charity Hospital. Dr. Miner was an active and influential member of the city and county medical societies, of the State Medical Society and of the American Medical Association, and always attracted much attention by the force and originality of his views and methods, which were, however, always practical and conservative. He delivered his last course of lectures in the session of 1881–82. When in the midst of his active and useful career he was stricken with paralysis, and since then has mostly been confined to his house, and for the last three years to his bed. For many years previous to this, his locomotion had been considerably impaired, but with steady hand and resolute will he continued to do an immense amount of labor and probably had as large a surgical practice as any person could possibly perform. He possessed that property, which is called personal magnetism to a remarkable degree, he had many office students, and the relation that existed between him and them, as well as with the students at the Medical College, was paternal and filial to a remarkable degree. "His boys" as he delighted to call them were devotedly attached to him, and his professional sons in all sections of this broad land will mourn as for a father gone when they learn of his demise. Gentlemen of the Erie County Medical Society; we have been called to part with a member and an associate who was an honor to us, and an honor and a great staff to the community in which he lived. Let his memory live in our hearts. Let us spread a record of our love and esteem upon our minutes. Let us send to his bereaved family our most heartfelt sympathy and condolence and let us attend his funeral in a body.

Dr. Rochester said that, in his brief memorial, he had tried to write in such a manner as would have pleased Dr. Miner himself had he been alive. "He frequently expressed himself to me," said the speaker, "as strongly against the reading of long memorials and eulogiums on occasions like this. So I have tried to sketch briefly only the salient points of his character."

Dr. Edward C. W. O'Brien, who was one of the dead surgeon's closest companions and most intimate friends, spoke as follows:

*Mr. Chairman:*—Among all the members of our profession in this city, probably there are not many who had the honor of a more intimate acquaintance with Dr. Miner than myself. If not, strictly speaking, one of my early preceptors, he was my friend from early manhood, one to whom I have been, and am, most deeply indebted; who gave me invaluable advice, not only in regard to the duties which would devolve upon me in the path of life I had chosen, but counselled me on general affairs, even, I had almost said as a father would counsel a son. Always accessible, never repellant, ever willing to give from his bountiful store of knowledge, to those less favored than himself, he may truly be said to have been the young man's—particularly the young physician's—friend; by whom, in turn, he was truly loved and highly respected. Dr. Miner fully deserved the brilliant professional reputation he had attained. A man of great natural ability, of deep study, and of wide experience; it is no wonder that he came to be looked upon as one of the bright and shining lights of the medical profession. Noble in character and presence, he always commanded attention and admiration in whatever circle he appeared. His reputation as a surgeon had become national long before he was overtaken by his disabling illness, and his remarkable skill and wisdom were so well known that hardly a day passed, before he was stricken down, without a call upon him by even the ablest members of our profession in this portion of the state for aid and advice in cases likely to baffle human skill. His charity was large, his friendship was sincere and his heart was big and honest. He gave freely to the poor—never turning a deaf ear to the cry of distress, or refusing his professional aid to the stricken and needy. His sense of honor was as fine and keen as that of any man who ever won a medical diploma. This trait of Dr. Miner was so well known and thoroughly appreciated as to frequently call forth the remark: "Dr. Miner could not be dishonorable under any circumstances."

Fortunate, indeed, will be the physician or surgeon who may in the future, so nobly win, and so richly deserve the admiration, confidence and affection of the local members of the medical fraternity as did Julius F. Miner.

Dr. Lucien F. Howe said that on many similar occasions the old admonition to say nothing of the dead but good might well be altered for the sake of truthfulness to *nil de mortuis nisi verum.* But for Dr. Miner, fortunately, both the maxims can be harmonized with honest severity. Regarding him from the standpoint of a surgeon, he could be spoken of as one of the oldest, as he was one of the most successful and prominent in the profession. His identity with the two large hospitals and a medical college of this city, if nothing else, would be sufficient to make him eminent. These institutions, which he either helped to found or so greatly strengthened with his zeal and untiring energy that they remain to-day, are the lengthened shadow of the man who is gone. His work as editor on the Buffalo Medical and Surgical Journal made the paper the exponent of professional thought for this section of the country. Dr. Miner's strength of character and untiring energy were told of and it was said that to the honor of the man, in medicine he was venerated as an authority. With his dominating characteristics it may be said truthfully that he can appear before the bar above, standing there like an honest man, with no mask upon his face and no shackles on his conscience.

Dr. John Cronyn, who had been associated with him in work at the Sisters's Hospital for years, spoke in a most feelingly and tender manner of his former colleague.

Remarks were also made by Dr. P. H. Strong, Dr. William Ring, Dr. Wall, Dr. Harrington, Dr. J. C. Greene, Dr. Phelps, Dr. Barnes, Dr. Bartlett and others.

The society attended the funeral in a body. There survives him his loving and faithful wife, a married daughter and a son, Mr. Worthington C. Miner, of this city, a lawyer of marked ability.

We are glad to be able to present to our readers this fine engraving of the Doctor, which will be treasured by many who were wont, in their college days, to look upon Dr. Miner, as their trusty friend, and who are yet proud to remember that they were, as he was accustomed to designate them, his "boys."

It is sad to reflect that the men who made the college with which they were connected famous, have, one by one, departed to the great majority, and of them all our distinguished friend Dr. Rochester alone remains.

Who will take their places?

## Figures and Tables

**Figure f1:**